# Human mesenchymal stem cells maintain their phenotype, multipotentiality, and genetic stability when cultured using a defined xeno-free human plasma fraction

**DOI:** 10.1186/s13287-017-0552-z

**Published:** 2017-04-27

**Authors:** Arantxa Blázquez-Prunera, José María Díez, Rodrigo Gajardo, Salvador Grancha

**Affiliations:** 1 Research and Development, Bioscience Industrial Group, Grifols, Parets del Vallès, Barcelona Spain; 2 Faculdade de Engenharia, Universidade do Porto, Porto, Portugal; 3 Cell Culture and Virology Laboratory, Research & Development Biologics, Industrial Group, Grifols, Carrer Llevant, 11, 08150 Parets del Vallès, Barcelona Spain

**Keywords:** Xeno-free, Mesenchymal stem cells, Culture, Human plasma fraction, Multipotentiality, Genetic stability

## Abstract

**Background:**

Mesenchymal stem cells (MSCs) show promising characteristics for their use in advanced therapy medicinal products. However, there are some unresolved concerns, such as the use of animal components for their expansion.

In this study we assessed the suitability of a xeno-free supplement for cell culture (SCC) derived from human plasma, to culture and expand human MSCs (hMSCs) from different origins. Characteristics of viable cultured hMSCs such as genetic stability, phenotype and multipotentiality were qualitatively evaluated.

**Methods:**

hMSCs from adipose tissue (AT), bone marrow (BM) and umbilical cord (UC) and supplier sources (commercial/non-commercial) were used. After hMSCs expansion in a xeno-free medium, classical hMSCs markers were studied by immunocytochemistry, and genetic stability was tested by classic karyotyping. The capacity of hMSCs to differentiate into adipogenic, osteogenic, and chondrogenic cells in differentiation media was assessed using different staining. Different lots of SCC were used to assure consistency between batches.

**Results:**

All hMSCs tested maintained their morphology and adherence to plastic during their expansion, and preserved their genetic stability, phenotype and differentiation potential. No differences were observed when using different lots of SCC. Moreover, the proliferation rate, evaluated as population doubling time (PDT) of commercial BM and AT hMSCs, was higher in the xeno-free medium than in the control media provided by the suppliers of the cells (PDT of 4.6 for BM-hMSC and 6.4 for AT-hMSC in xeno-free medium, and 7.0 and 14.7 respectively in the commercial media). UC-hMSCs PDT was similar in all the media tested. When using non-commercial BM-hMSCs, PDT was lower in the xeno-free medium, but reverted to the control level with the addition of growth factors.

**Conclusions:**

SCC-containing medium can be a feasible xeno-free alternative to expand hMSCs for advanced therapies.

## Background

Mesenchymal stem cells (MSCs) are a subset of non-hematopoietic adult multipotent cells originating from the mesoderm that can be isolated from almost all tissues and expanded in vitro [1]. MSCs are defined by their multipotentiality, with the ability to differentiate into adipocytes, osteoblasts and chondrocytes [2].

Human MSCs (hMSCs) are a promising tool in regenerative medicine and as a treatment for immune-mediated diseases [3]. However, there are limitations that need to be addressed to determine the safety of use in humans. One of the main concerns is related to the use of fetal bovine serum (FBS) as a supplement in cell culture medium to expand hMSCs. The introduction of animal derivatives into human cell cultures is not recommended since animal proteins can become associated with MSCs, and thus induce an immune rejection to the host [4]. Another drawback is the lack of lot-to-lot consistency of the FBS and its limited source [5–7]. For these reasons, the presence of animal components is discouraged in the culture media used to expand hMSC for therapy [4].

The most commonly used FBS-free options are the serum-free media, chemically defined media, and human serum- and platelet lysate-supplemented media [8, 9]. Serum-free media does not contain serum; however, it may contain proteins derived from animals, such as albumin, hormones, and attachment factors. Thus, it may not be a xeno-free medium. In the chemically defined media, all the components and concentrations are known; proteins are obtained from recombinant bacteria or are chemically synthesized, being entirely free of animal-derived components. These media do not present batch-to-batch variations [8, 10].

Although different attempts have been made to develop a chemically defined media, most of them have limitations, such as only supporting expansion for a single passage or at a low proliferation rate [11, 12]. Some promising chemically defined media are already on the market. One is the FDA-approved StemPro MSC SFM from Invitrogen (Carlsbad, CA, USA), which allows hMSCs isolation and expansion. However, some drawbacks regarding the use of this medium are evident, such as different expression levels of some molecules and differentiation potential, when compared with hMSCs expanded in an FBS-supplemented medium [9]. Other commercially available xeno-free media also present controversial results regarding the expansion of hMSCs from different origins, hMSCs attachment or proliferation rates [13–24]. Among the different xeno-free/serum-free options that are currently in use, the most common ones are the human platelet lysates and the chemically defined serum-free media [14, 15, 25–28]. Furthermore, the use of supplement for cell culture (SCC) derived from human plasma showed promising results in bone marrow (BM) hMSCs expansion, preserving not only hMSC typical characteristics and multipotentiality [29], but also hMSC immunomodulatory properties and chemotaxis [30].

In this study, SCC was used to culture and expand hMSCs isolated from different origins (BM, umbilical cord [UC], and adipose tissue [AT]) and supplier sources (commercial/non-commercial). To assess the suitability of SCC to expand hMSCs, cell proliferation, adherence, genetic stability, typical markers and multipotentiality were evaluated.

## Methods

### Objective and study design

The objective of this study was to assess whether xeno-free SCC is suitable for culture and expansion of hMSCs isolated from BM, AT and UC, using different lots of SCC to confirm consistency between batches. Characteristics of viable cultured hMSCs such as genetic stability (normal karyotype), phenotype (typical surface markers expression) and multipotentiality (adipogenic, osteogenic and chondrogenic differentiation potential) were qualitatively evaluated.

### hMSCs used

Different hMSC lines obtained from BM, UC and AT were acquired from two different suppliers (Lonza Group Ltd, Basel, Switzerland, and Promocell GmbH, Heidelberg, Germany). Ethics approval was not required for use of commercial cells. Furthermore, non-commercial BM-hMSCs were kindly supplied by Inbiobank and the Instituto Nacional de Engenheria Biomedica (INEB, Porto, Portugal). Inbiobank isolated the cells following manufacturing procedures based on ISO9001:2000 under Good Manufacturing Practice (GMP) conditions. INEB cells were obtained from discarded bone tissues of two different patients at the Hospital São João, Porto, who provided written consent. Confidentiality of donors’ information was guaranteed. hMSCs were isolated using DMEM supplemented with FBS. Ten per cent DMSO was added to the isolated hMSCs, which were frozen at -80 °C overnight before being transferred to a liquid nitrogen tank. All hMSCs stocks were kept in a liquid nitrogen tank until their use.

### Xeno-free medium

As a xeno-free substitute of FBS, a human plasma derivative (cell culture supplement, SCC, Grifols, Barcelona, Spain) was used. This product is derived from human plasma specifically collected as starting material for the industrial production of different plasma therapeutic proteins. SCC is obtained through cold ethanol industrial plasma fractionation, and it contains a stable and defined fraction of human proteins from plasma pools which contain samples from at least 1000 different healthy donors [29]. Due to the large number of donors in each pool, a high consistency among lots is expected. SCC is manufactured following GMP procedures. Each plasma donation is tested for the presence of pathogen agents (transfusion transmissible). Furthermore, a dedicated step with viral inactivation capacity (gamma irradiation) is included in the manufacturing process, in addition to the various steps taken to eliminate/remove pathogens [29].

The xeno-free medium used consists of Dulbecco’s modification of Eagle’s medium F12 (ref. 21221, Gibco-Life Technologies, Carlsbad, CA, USA) supplemented with SCC and other growth factors from platelet lysate (PL-Medium) as described elsewhere [29]. The platelet lysate used was obtained through the freeze-thaw method [31]. In this study, a variation without the addition of platelet lysate (XF-Medium) was also used.

### hMSC cell culture and growth evaluation

All hMSCs were thawed in Reference Medium; for commercial hMSCs, MSC basal medium (ref. PT3238, Lonza) or MSC cell growth medium (ref. C-28010, PromoCell) were used depending on the hMSCs commercial supplier. Non-commercial hMSCs were thawed in FBS-Medium, composed of DMEM low glucose (ref. 21969, Gibco-Life Technologies) supplemented with 10% MSC-qualified FBS (ref. 10500, Gibco-Life Technologies), 1% L-glutamine (ref. 25030, Gibco-Life Technologies) and 1% penicillin/streptomycin (ref. 15140, Gibco-Life Technologies). hMSCs were cultured at 37 °C and 8% CO_2_. Medium was replaced every 3–4 days until arriving at 80% confluence, when cells were detached using xeno-free trypsin (Tryple Express, ref. 12604, Gibco-Life Technologies). hMSCs were split and seeded at a cell density of 5000–6000 cells/cm^2^ using the different media (Reference Medium, XF-Medium lot 1 and lot 2, PL-Medium lot 1 and lot 2). After each passage, population doubling time (PDT) was calculated as described elsewhere [29]. Statistical analysis was performed using GraphPad Prism Version 5.01 (GraphPad Software, San Diego, CA, USA) and applying the non-parametric Kruskal-Wallis test followed by Dunn’s multiple comparison test.

### hMSC genetic stability under culture

To determine if xeno-free-expanded hMSCs were genetically stable, hMSCs cultures at early and late passages were compared. UC-hMSCs and AT-hMSCs were thawed in Reference Medium and seeded in three flasks at a cell density of 5000 cells/cm^2^. After 24 hours the medium was replaced by new Reference Medium, XF-Medium or PL-Medium. After 3–4 passages, a new vial of the same cell line and lot was thawed following the same protocol. When cultures were around 60% confluence, flasks were analysed by GTGbanding. Chromosomes were identified and analysed according to ECA Cytoteogenetic Guidelines.

### hMSC phenotypic characterization

The xeno-free-expanded hMSCs phenotype was evaluated by immunofluorescent staining of typical hMSCs surface markers as previously described [29]. Two negative markers - CD14 (ref. MAB1219, Merck Millipore, Billerica, MA, USA) and CD19 (ref. MAB1794, Merck Millipore) – and seven positive markers – CD29 (ref. 303002, Biolegend, San Diego, CA, USA) , CD44 (ref. CBL154, Merck Millipore), CD73 (ref. 344004, Biolegend), CD90 (ref. CBL415, Merck Millipore), CD105 (ref. MABT117, Merck Millipore), CD166 (ref. 343902, Biolegend), Stro-1 (ref. MAB4315, Merck Millipore) - were studied.

Cells were fixed with Intracellular (IC) Fixation Buffer (ref. FB001, Invitrogen-Life Technologies, Carlsbad, CA, USA) and incubated at room temperature (RT) with blocking solution. Then cells were incubated overnight at 4 °C with the primary antibodies (a 1:100 dilution in blocking solution was used for CD14, CD19, CD29, CD44, Stro-1; a 1:50 for CD90 and 1:20 for CD73, CD105, CD166). After incubation, cells were washed twice with PBS and twice with blocking solution. After the incubation at RT with blocking solution, the secondary antibodies were added to the plate (donkey anti-mouse IgG conjugated with FITC (ref. AP192F, Merck Millipore) and goat anti-mouse IgM conjugated with Cy3 (ref. AP128C, Merck Millipore). After washing the cells with PBS, samples were counterstained with 4’,6-diamidino-2-phenylindole (DAPI, ref. D3571, Invitrogen-Life Technologies) and visualized under a fluorescent microscope (Axiobserver LD Plan-Neofluar objective; Carl Zeiss, Jena, Germany).

### Osteogenic differentiation

BM-hMSCs (commercial and non-commercial), AT-hMSCs and UC-hMSCs were expanded in the different media. After reaching 80% confluence, cells were harvested and 6 × 10^4^/well hMSCs were seeded in a 24-well plate. Osteogenesis was induced by commercial osteogenic medium (ref. C-28013, Promocell). After 7 days of incubation, some of the samples were fixed and the alkaline phosphatase activity was stained with nitro-blue tetrazolium chloride and 5-bromo-4-chloro-3’-indolyl phosphate salt and nitro-blue tetrazolium chloride (BCIP/NBT tablet, ref. B5655, Sigma-Aldrich, St. Louis, MO, USA). After 21 days of incubation, the presence of extracellular calcium deposits was assessed by its specific staining with Alizarin Red S (ref. A5533, Sigma-Aldrich) to determine osteogenic differentiation.

### Chondrogenic differentiation

BM-hMSCs (commercial and non-commercial), AT-hMSCs and UC-hMSCs were expanded in the different media. After reaching 80% confluence, 3 × 10^5^ cells were placed in a 15-mL conical tube. A first centrifugation at 150 g over 15 minutes was done in the expansion media. After that, the media were replaced by commercial chondrogenic differentiation medium (ref. C-28012, Promocell) and tubes were centrifuged at 150 g for 5 minutes. Without disturbing the pellets, tubes were incubated at 37 °C 5% CO_2_ and spheres were allowed to form. After 21 days in culture, spheres were fixed with IC Fixation Buffer for 1 hour and cartilage was stained with Alcian Blue (ref. A3157, Sigma-Aldrich) to determine chondrogenic differentiation.

### Adipogenic differentiation

BM-hMSCs (commercial and non-commercial), AT-hMSCs and UC-hMSCs were expanded in the different media. After reaching 80% confluence, cells were harvested and 6 × 10^4^/well hMSCs were seeded in a 24-well plate. Adipogenesis was induced by commercial adipogenic medium (ref. C-28011, Promocell). After 14 days incubation, samples were fixed and lipid droplets were stained with Oil Red O (ref. O0625, Sigma-Aldrich) to determine adipogenic differentiation.

## Results

### hMSc growth in xeno-free conditions

**Fig. 1:**
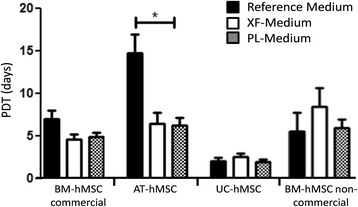
Population doubling time (*PDT*) of different human mesenchymal stem cells. Bone marrow (*BM*)-, adipose tissue (*AT*)- and umbilical cord (*UC*)-derived human mesenchymal stem cells (*hMSCs*) in Reference media (Commercial medium 1, Commercial Medium 2, FBS-Medium), XF-Medium and PL-Medium (*n* = 3–22. ^*^Denotes *p* < 0.05)

BM-hMSCs (commercial and non-commercial), AT-hMSCs and UC-hMSCs could be expanded in xeno-free medium, with (PL-Medium) and without (XF-Medium) platelet lysate. All hMSCs tested adhered to the plastic surface of the culture flask without additional supplementation with attachment factors or surface coating. No differences were observed among the different lots of SCC used. As seen in Fig. 1, commercial BM-hMSCs showed a tendency to grow faster in xeno-free medium (PDT mean = 4.6) than in Reference Medium (PDT mean = 7). AT-hMSCs grew faster in xeno-free medium (PDT mean = 6.4) than in the commercial Reference Medium (PDT mean = 14.7). The addition of platelet lysate into the xeno-free medium did not produce significant changes in the PDT of the commercial hMSCs lines (BM-hMSC = 4.8, AT-hMSC = 6.2, UC-hMSC = 1.9). The cell replicative capacity of UC-hMSCs was similar in all tested media (XF = 2.5, PL = 1.9, Reference = 2). Non-commercial BM-hMSCs grew slower in XF-Medium (PDT mean = 8.3) than in the Reference Medium (FBS-Medium, PDT mean = 5.5); however, the cell replicative capacity was equivalent to the control in Reference Medium when growth factors from platelet lysate were added into the xeno-free medium (PDT mean = 5.5).

### hMSC genetic stability

**Fig. 2:**
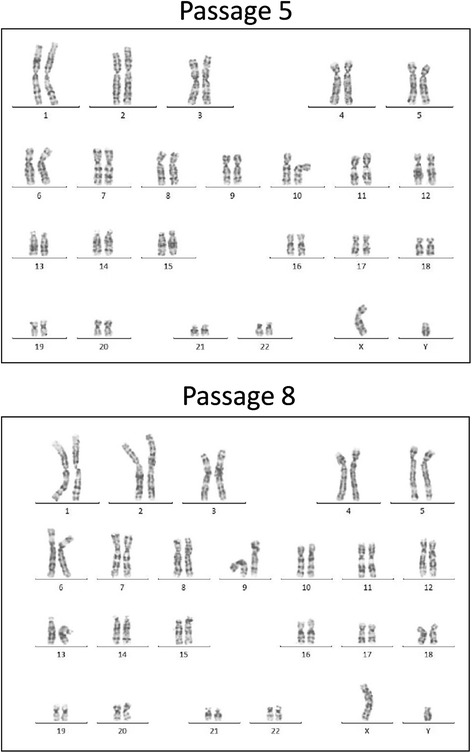
Genetic stability analysis of xeno-free expanded human mesenchymal stem cells after long-term culture. Representative karyotypes of just-thawed umbilical cord-human mesenchymal stem cells in Reference Medium (Passage 5) and long-term culture in xeno-free medium (Passage 8)

To determine whether the faster hMSCs growth rate in xeno-free medium fails to induce chromosomal aberrations, hMSCs were cultured throughout different passages and their genomic stability was assessed using conventional cytogenetic analysis. All the karyotypes obtained showed a normal diploid karyotype. hMSCs were genetically stable after 3 and 4 passages in xeno-free medium culture (with and without growth factors from platelet lysate) (Fig. 2).

### Expression of typical hMSC surface markers

**Fig. 3:**
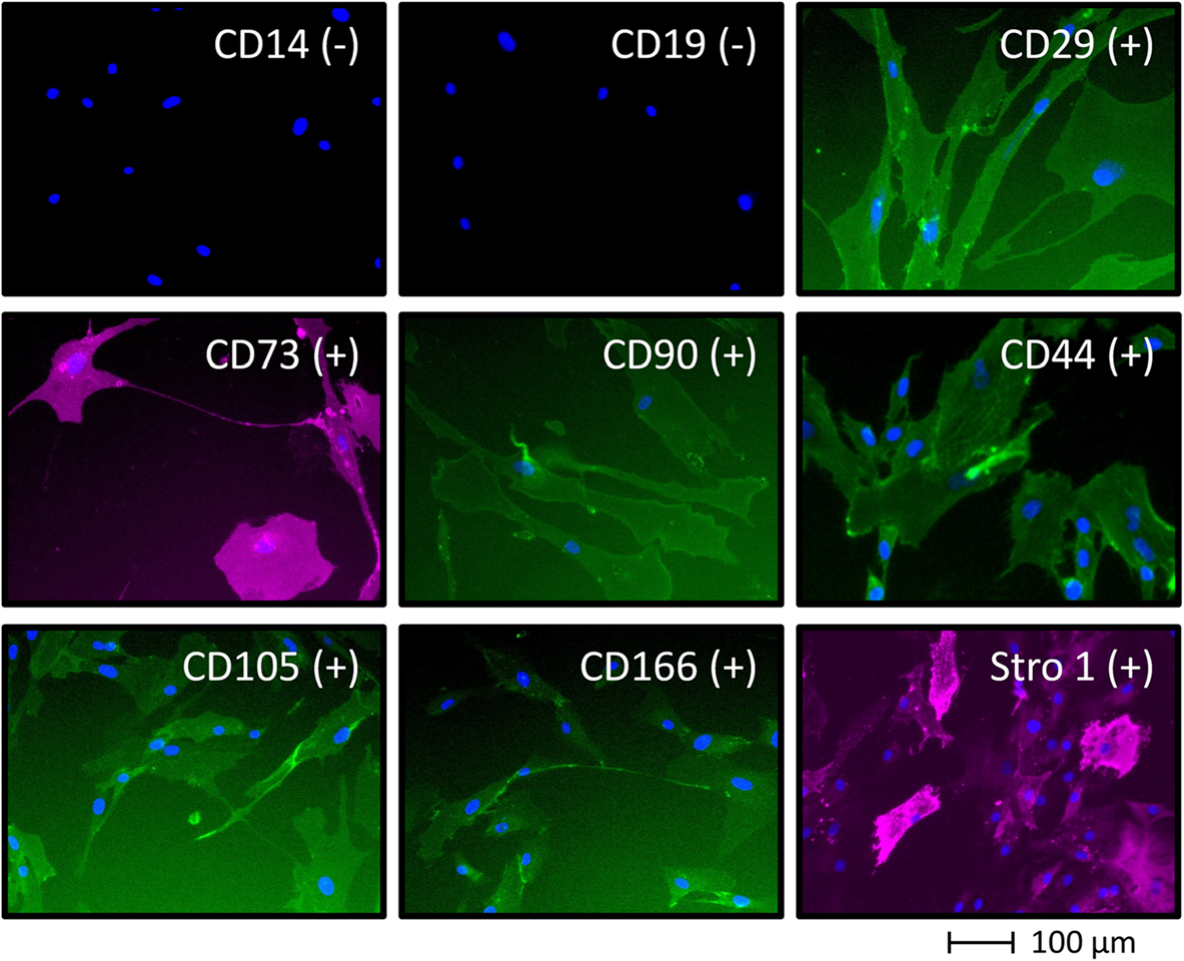
Phenotypic characterization of human mesenchymal stem cells. Expression of the typical human mesenchymal stem cells surface markers by xeno-free expanded human mesenchymal stem cells as determined by immunofluorescence staining (representative images).

BM-hMSCs (commercial and non-commercial), AT-hMSCs and UC-hMSCs expanded in the different xeno-free media (XF-Medium lot 1, XF-Medium lot 2, PL-Medium lot 1, PL-Medium lot 2) presented the normal hMSC phenotype, being negative for CD14, CD19 and positive for CD29, CD44, CD73, CD90, CD105, CD166 and Stro-1 (Fig. 3).

### Multipotentiality of xeno-free-expanded hMSC

#### Osteogenic differentiation

**Fig. 4:**
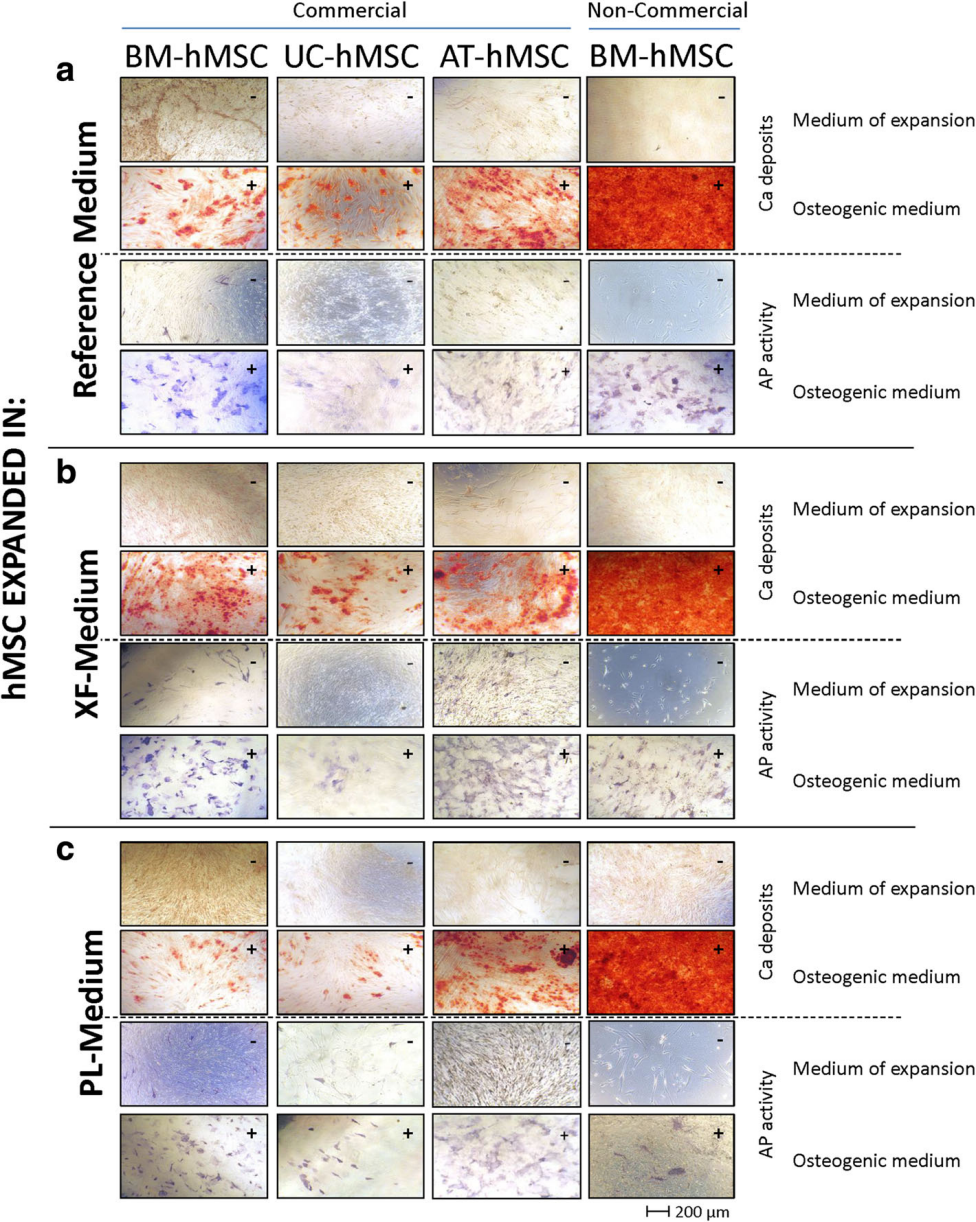
Osteogenic differentiation of human mesenchymal stem cells. Representative images of commercial and non-commercial bone marrow (*BM*)-, adipose tissue (*AT*)- and umbilical cord (*UC*)-derived human mesenchymal stem cells (*hMSC*s) expanded in Reference Medium, and xeno-free medium with and without the growth factors from platelet lysate (PL-Medium, XF-Medium). hMSCs were cultured for 21 days in osteogenic differentiation medium or the medium of expansion. After 7 days, the alkaline phosphatase (*AP*) activity was stained in *blue* with BCIP/NBT. After 21 days, the extracellular calcium (*Ca*) deposits was stained with Alizarin Red S in *red*

BM-hMSCs (commercial and non-commercial), AT-hMSCs and UC-hMSCs expanded in the different xeno-free media (XF-Medium lot 1, XF-Medium lot 2, PL-Medium lot 1, PL-Medium lot 2) could be differentiated into osteoblasts (Fig. 4). After 21 days in osteogenic medium, hMSCs showed the typical cuboidal and flattened osteoblastic morphology. Extracellular calcium deposits and elevated alkaline phosphatase activity were observed in the differentiated hMSCs. Some differences could be observed when comparing the different cell lines; non-commercial BM-hMSCs presented the highest level of osteogenic differentiation, and UC-hMSCs presented the lowest level of differentiation. No substantial differences were observed regarding the media used to expand the hMSCs or the lots of SCC tested.

#### Chondrogenic differentiation

**Fig. 5:**
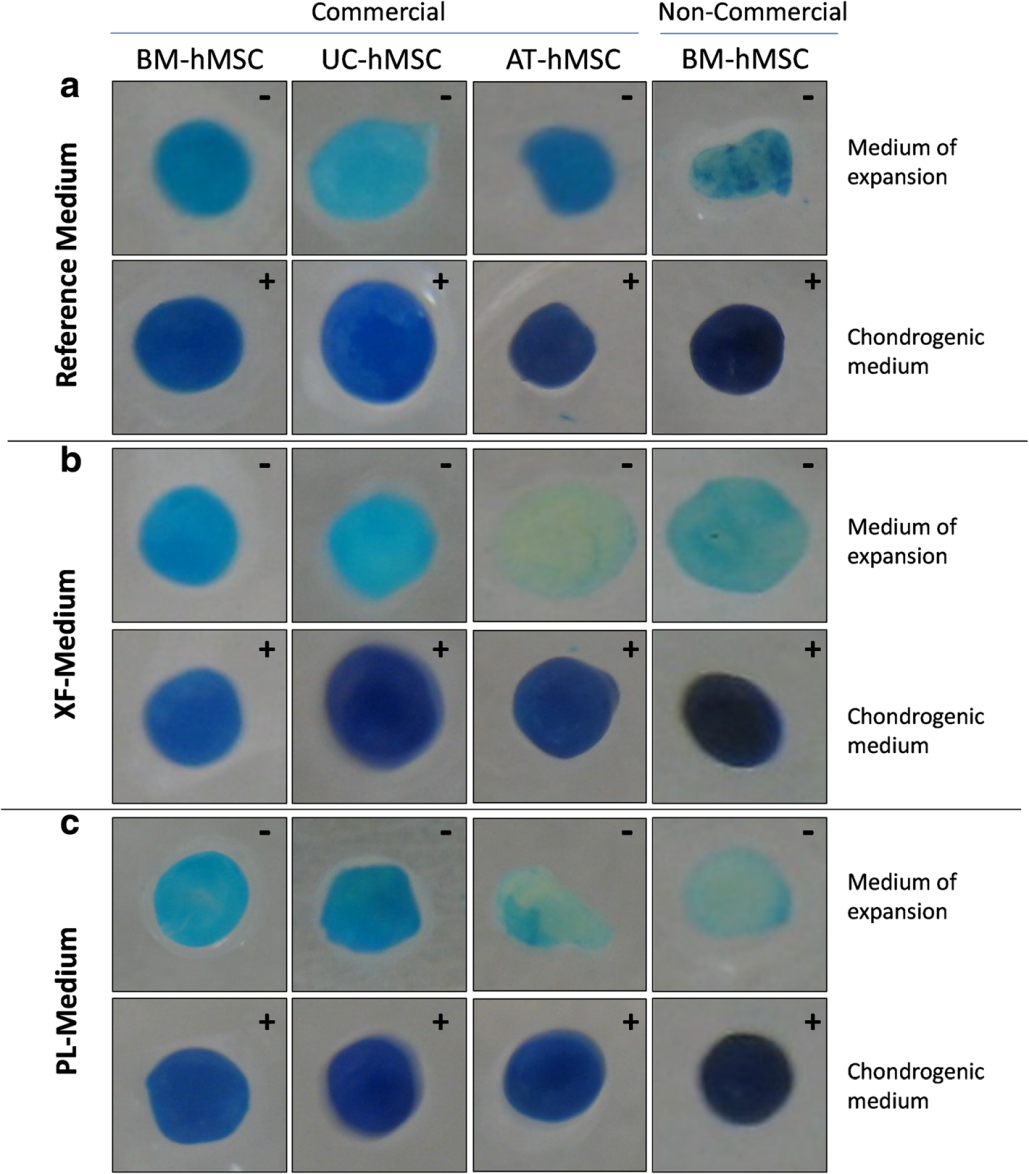
Chondrogenic differentiation of human mesenchymal stem cells. Representative images of commercial and non-commercial bone marrow (*BM*)-, adipose tissue (*AT*)- and umbilical cord (*UC*)- derived human mesenchymal stem cells (*hMSCs*) expanded in Reference Medium, and xeno-free medium with and without the growth factors from platelet lysate (PL-Medium, XF-Medium). hMSCs were cultured for 21 days in chondrogenic differentiation medium or the medium of expansion. Alcian Blue was used to stain the extracellular cartilage matrix in *dark blue*

BM-hMSCs (commercial and non-commercial), AT-hMSCs and UC-hMSCs expanded in the different xeno-free media (XF-Medium lot 1, XF-Medium lot 2, PL-Medium lot 1, PL-Medium lot 2) preserved their potential of differentiation into cartilage (Fig. 5). After 21 days in chondrogenic medium, the typical glycosaminoglycans present in cartilage could be stained with Alcian Blue. In all commercial hMSCs types and origins, expanded in the different media and lots of SCC, chondrogenic differentiation was observed at a similar level. Non-commercial BM-hMSCs presented a higher presence of glycosaminoglycans than the commercial hMSCs.

#### Adipogenic differentiation

**Fig. 6:**
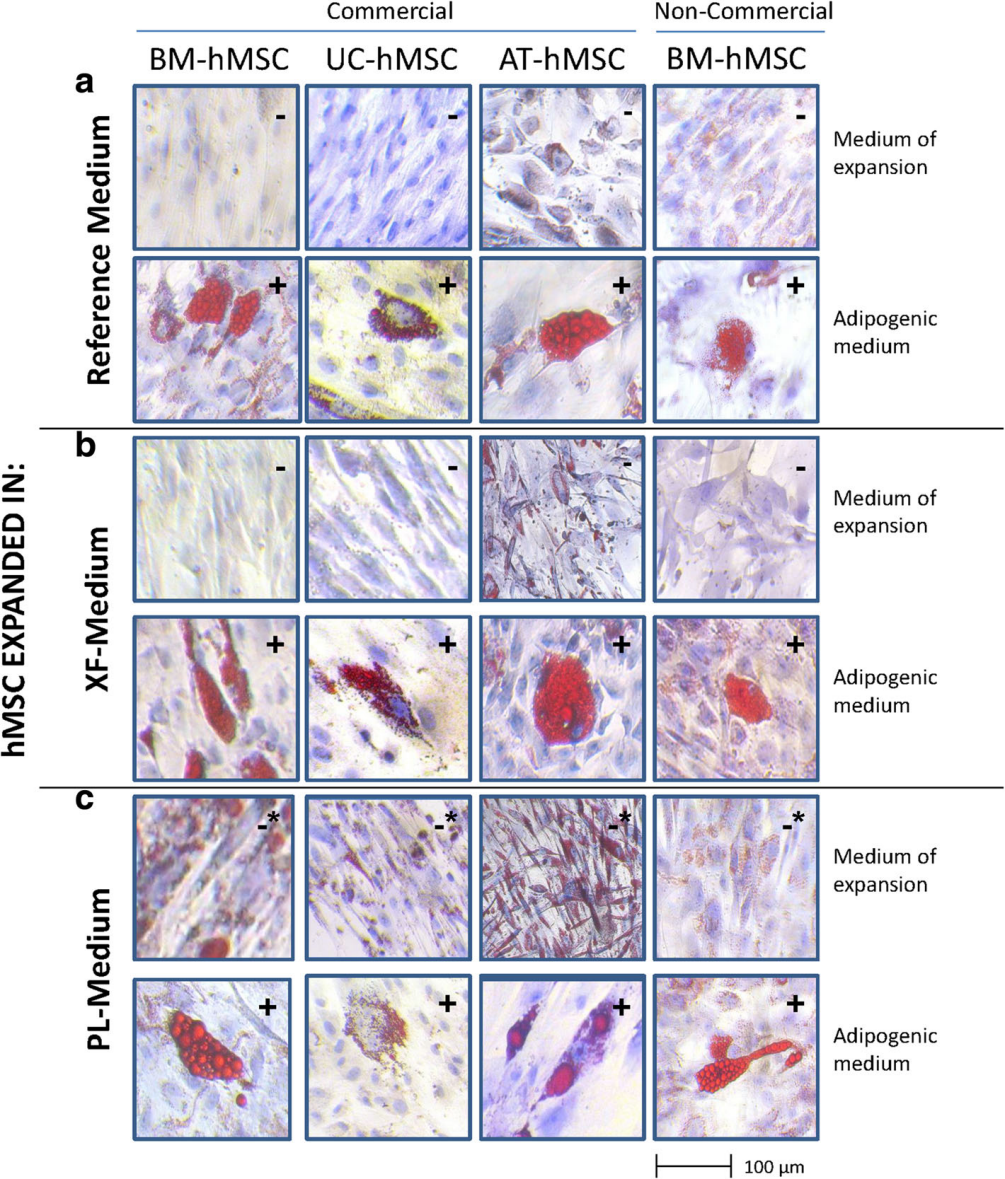
Adipogenic differentiation of human mesenchymal stem cells. Representative images of commercial and non-commercial bone marrow (*BM*)-, adipose tissue (*AT*)- and umbilical cord (*UC*)- derived human mesenchymal stem cells (*hMSCs*) expanded in Reference Medium, and xeno-free medium with and without platelet lysate (PL-Medium, XF-Medium). hMSCs were cultured for 14 days in adipogenic differentiation medium or the medium of expansion. Lipid vesicles in adipocytes were stained with Oil Red O and are seen in *red*

BM-hMSCs (commercial and non-commercial), AT-hMSCs and UC-hMSCs expanded in the different xeno-free media (XF-Medium lot 1, XF-Medium lot 2, PL-Medium lot 1, PL-Medium lot 2) could be differentiated into adipocytes (Fig. 6). After 14 days in adipogenic medium, characteristic lipid droplets could be stained with Oil Red O. No differences were observed among the adipogenic differentiation of the tested cell types and origins, media or lots of SCC.

## Discussion

hMSCs therapies are of great interest, not only for their multipotent characteristics, but also for their use in treating immune diseases [3]. In regenerative medicine, there are concerns regarding the immune rejection that a patient can suffer if animal proteins become adhered to the in vitro expanded human cells [4, 7]. Thus, the replacement of the commonly used FBS is a necessity, and different strategies are being developed. In a previous report, we described that commercial BM-hMSCs could be expanded in SCC-containing medium supplemented with growth factors from platelet lysate (PL-Medium) [29]. Moreover, hMSCs expanded in SCC-based medium have been shown to preserve their immunomodulatory and chemotactic properties [30]. In this study, we extended the results for non-commercial BM-hMSCs and commercial AT-hMSCs and UC-hMSCs. Our results indicated that the use of platelet lysate as a supplement in SCC-containing medium is optional. In addition, we showed that SCC can be used to culture and expand hMSCs under xeno-free conditions, maintaining their phenotype, genetic stability and multipotentiality.

Xeno-free media supplemented with human autologous serum has been shown to support hMSCs expansion; however, it would be challenging to obtain an adequate amount of serum to expand hMSCs sufficiently, and the age of the donor would influence its properties [8, 32]. There is controversy on the use of this medium, and contradictory results have been published [33–41]. There are no established protocols for the isolation and culture of hMSCs; thus, different laboratories use different strategies, making it difficult to compare the outcomes of different studies that try to develop a xeno-free medium. Usually, the strategies consist of growing hMSCs in the media in the pipeline and checking the International Society for Cellular Therapy hMSCs defining characteristics (hMSCs adherence to plastic, phenotype, and differentiation into osteoblasts, chondroblasts and adipocytes) [42].

SCC is an industrial GMP product, obtained from a large quantity of plasma sourced from more than 1000 donors, which considerably reduces the lot-to-lot variability. Moreover, no supply problems are expected. In our study, all hMSCs tested were able to adhere and be expanded in XF-Medium and PL-Medium. The proliferation rate of the commercial BM- and AT- hMSCs was higher than in the Reference Medium, as observed in the previous studies [42]. As a high proliferation rate could induce chromosomic aberrations, the genetic stability of the cultures was assessed. hMSCs were passaged for 3 and 4 runs in XF-Medium, PL-Medium and Reference Medium. Our results showed that hMSCs expanded using the different media were genetically stable, showing a normal diploid karyotype. Other studies also showed a higher proliferation of hMSCs in other xeno-free and serum-free media than in FBS-supplemented media; however, usually the genetic stability was not studied and only hMSCs from one origin were used [12, 13, 41].

After verifying that the cells could be expanded in the xeno-free media, we tested whether the defining hMSCs characteristics were maintained. All hMSCs used presented the typical hMSC phenotype, being positive for CD29, CD44, CD73, CD90, CD105, CD166 and Stro-1, and negative for CD14, CD19. The multipotentiality of the cells was also studied; all the cells tested could be differentiated into osteoblasts, chondrocytes and adipocytes. Although the assays performed were qualitative, some differences in the differentiation levels could be observed when using the different cell types. Non-commercial BM-hMSCs showed a higher chondrogenic and osteogenic differentiation than the other cell types, an observation that was made in other studies [24]. On the other hand, UC-hMSCs showed a lower osteogenic differentiation, when compared with the other cells, which was also observed by other researchers [43, 44].

Although platelet lysate has shown promising characteristics for hMSCs culture and expansion and is being used in many studies, some drawbacks are coming out, such as donor variability [45], decreased expression of adipogenic and osteogenic differentiation markers [46, 47], and interaction with immunomodulatory properties of MSCs [48, 49]. Due to these concerns, in this study we tested the use of SCC without the addition of platelet lysate. Our results showed that the addition of platelet lysate to SCC-containing Medium is optional. All the hMSCs defining characteristics were maintained in XF-Medium without the addition of platelet lysate. The only differences observed were in the proliferation rate of non-commercial BM-hMSCs, which was higher when introducing platelet lysate to the medium. Thus, due to the potential negative effects, as mentioned before, we recommend avoiding the supplementation with platelet lysate in SCC-supplemented media.

It has been previously shown that SCC can be used to culture not only hMSCs, but also Chinese hamster ovarian cells, Vero cells, and mouse BALB/c myeloma cells [29]. All the studies with the commercial cell lines were done in parallel using different lots of human plasma fraction. In this study, hMSCs behaved similarly regardless of the SCC lot used, which indicates that the batch selected does not present significant variations regarding the other batches, as expected from its origin (large plasma pools from over 1000 donors).

## Conclusions

SCC is a highly robust human plasma fraction. BM-hMSCs (commercial and non-commercial), AT-hMSCs and UC-hMSCs could be expanded in a xeno-free media including SCC as a supplement while maintaining their genetic stability and typical hMSCs phenotype and multipotentiality. Moreover, our results suggest that the platelet lysate-free composition is suitable for culture and expansion of hMSCs in xeno-free conditions for human cell therapies.

## Abbreviations

AT: Adipose tissue
BM: Bone marrow
FBS: Fetal bovine serum
GMP: Good Manufacturing Practice
hMSC: Human mesenchymal stem cell
MSC: Mesenchymal stem cell
PDT: Population doubling time
SCC: Supplement for cell culture
UC: Umbilical cord

## References

[1] Singer NG, Caplan AI (2011). Mesenchymal stem cells: mechanisms of inflammation. Annu Rev Pathol..

[2] Horwitz EM, Le Blanc K, Dominici M, Mueller I, Slaper-Cortenbach I, Marini FC, Deans RJ, Krause DS, Keating A, International Society for Cellular Therapy (2005). Clarification of the nomenclature for MSC: the International Society for Cellular Therapy position statement. Cytotherapy..

[3] Singec I, Janrial R, Crain A, Nikkhah G, Snyder EY (2007). The leading edge of stem cell therapeutics. Annu Rev Med..

[4] European Medicines Agency. Guideline on the use of bovine serum in the manufacture of human biological medicinal products. Committee for Medicinal Products for Human Use (CHMP) 2013. EMA/CHMP/BWP/457920/2012 rev 1 [http://www.ema.europa.eu/docs/en_GB/document_library/Scientific_guideline/2013/06/WC500143930.pdf].

[5] Aldahmash A, Haack-Sørensen M, Al-Nbaheen M, Harkness L, Abdallah BM, Kassem M (2011). Human serum is as efficient as fetal bovine serum in supporting proliferation and differentiation of human multipotent stromal (mesenchymal) stem cells in vitro and in vivo. Stem Cell Rev..

[6] Bieback K, Hecker A, Kocaömer A, Lannert H, Schallmoser K, Strunk D, Klüter H (2009). Human alternatives to fetal bovine serum for the expansion of mesenchymal stromal cells from bone marrow. Stem Cells..

[7] Spees JL, Gregory CA, Singh H, Tucker HA, Peister A, Lynch PJ, Hsu SC, Smith J, Prockop DJ (2004). Internalized antigens must be removed to prepare hypoimmunogenic mesenchymal stem cells for cell and gene therapy. Mol Ther..

[8] Jayme DW, Smith SR (2000). Media formulation options and manufacturing process controls to safeguard against introduction of animal origin contaminants in animal cell culture. Cytotechnology..

[9] Jung S, Panchalingam KM, Rosenberg L, Behie LA (2012). Ex vivo expansion of human mesenchymal stem cells in defined serum-free media. Stem Cells Int..

[10] Shenoy M (2007). Animal Biotechnology.

[11] Lennon DP, Haynesworth SE, Young RG, Dennis JE, Caplan AI (1995). A chemically defined medium supports in vitro proliferation and maintains the osteochondral potential of rat marrow-derived mesenchymal stem cells. Exp Cell Res..

[12] Parker AM, Shang H, Khurgel M, Katz A (2007). Low serum and serum-free culture of multipotential human adipose stem cells. Cytotherapy..

[13] Lindroos B, Boucher S, Chase L, Kuokkanen H, Huhtala H, Haataja R, Vemuri M, Suuronen R, Miettinen S (2009). Serum-free, xeno-free culture media maintain the proliferation rate and multipotentiality of adipose stem cells in vitro. Cytotherapy..

[14] Miwa H, Hashimoto Y, Tensho K, Wakitani S, Takagi M (2012). Xeno-free proliferation of human bone marrow mesenchymal stem cells. Cytotechnology..

[15] Schallmoser K, Rohde E, Reinisch A, Bartmann C, Thaler D, Drexler C, Obenauf AC, Lanzer G, Linkesch W, Strunk D (2008). Rapid largescale expansion of functional mesenchymal stem cells from unmanipulated bone marrow without animal serum. Tissue Eng Part C Methods..

[16] Liu CH, Wu ML, Hwang SM (2007). Optimization of serum free medium for cord blood mesenchymal stem cells. Biochem Eng J..

[17] Hartmann I, Hollweck T, Haffner S, Krebs M, Meiser B, Reichart B, Eissner G (2010). Umbilical cord tissue-derived mesenchymal stem cells grow best under GMP-compliant culture conditions and maintain their phenotypic and functional properties. J Immunol Methods..

[18] Oikonomopoulos A, van Deen WK, Manansala AR, Lacey PN, Tomakili TA, Ziman A, Hommes DW (2015). Optimization of human mesenchymal stem cell manufacturing: the effects of animal/xeno-free media. Sci Rep..

[19] Chase LG, Yang S, Zachar V, Yang Z, Lakshmipathy U, Bradford J, Boucher SE, Vemuri MC (2012). Development and characterization of a clinically compliant xeno-free culture medium in good manufacturing practice for human multipotent mesenchymal stem cells. Stem Cells Transl Med..

[20] Corotchi MC, Popa MA, Remes A, Sima LE, Gussi I, Lupu PM (2013). Isolation method and xeno-free culture conditions influence multipotent differentiation capacity of human Wharton’s jelly-derived mesenchymal stem cells. Stem Cell Res Ther..

[21] Patrikoski M, Juntunen M, Boucher S, Campbell A, Vemuri MC, Mannerström B, Miettinen S (2013). Development of fully defined xeno-free culture system for the preparation and propagation of cell therapy-compliant human adipose stem cells. Stem Cell Res Ther..

[22] Simoes IN, Boura JS, Dos Santos F, Andrade PZ, Cardoso CM, Gimble JM, da Silva CL, Cabral JM (2013). Human mesenchymal stem cells from the umbilical cord matrix: successful isolation and ex-vivo expansion using serum-/xeno-free culture media. Biotechnol J..

[23] Gstraunthaler G (2003). Alternatives to the use of fetal bovine serum: serum-free cell culture. ALTEX..

[24] Li CY, Wu XY, Tong JB, Yang XX, Zhao JL, Zheng QF, Zhao GB, Ma ZJ (2015). Comparative analysis of human mesenchymal stem cells from bone marrow and adipose tissue under xeno-free conditions for cell therapy. Stem Cell Res Ther..

[25] Doucet C, Ernou I, Zhang Y, Llense JR, Begot L, Holy X, Lataillade JJ (2005). Platelet lysates promote mesenchymal stem cell expansion: a safety substitute for animal serum in cell-based therapy applications. J Cell Physiol..

[26] Schallmoser K, Bartmann C, Rohde E, Reinisch A, Kashofer K, Stadelmeyer E, Drexler C, Lanzer G, Linkesch W, Strunk D (2007). Human platelet lysate can replace fetal bovine serum for clinical-scale expansion of functional mesenchymal stromal cells. Transfusion..

[27] Cohn EJ, Strong LE (1946). Preparation and properties of serum and plasma proteins; a system for the separation into fractions of the protein and lipoprotein components of biological tissues and fluids. J Am Chem Soc..

[28] Lange C, Cakiroglu F, Spiess AN, Cappallo-Obermann H, Dierlamm J, Zander AR (2007). Accelerated and safe expansion of human mesenchymal stromal cells in animal serum-free medium for transplantation and regenerative medicine. J Cell Physiol..

[29] Díez JM, Bauman E, Gajardo R, Jorquera JI (2015). Culture of human mesenchymal stem cells using a candidate pharmaceutical grade xeno-free cell culture supplement derived from industrial human plasma pools. Stem Cell Res Ther..

[30] Blázquez-Prunera A, Almeida CR, Barbosa MA. Human bone marrow mesenchymal stem/stromal cells preserve their immunomodulatory and chemotactic properties when expanded in a human plasma-derived xeno-free medium. Stem Cells International. 2017 (in press). 10.1155/2017/2185351 PMC5446864 28588620

[31] Schallmoser K, Strunk D (2009). Preparation of pooled human platelet lysate (pHPL) as an efficient supplement for animal serum-free human stem cell cultures. J Vis Exp..

[32] Tunaitis V, Borutinskaité V, Navakauskiené R, Treigytė G, Ungurytė A, Aldonytė R, Magnusson KE, Pivoriūnas A (2011). Effects of different sera on adipose tissue-derived mesenchymal stromal cells. J Tissue Eng Regen Med..

[33] Dahl JA, Duggal S, Coulston N, Millar D, Melki J, Shahdadfar A, Brinchmann JE, Collas P (2008). Genetic and epigenetic instability of human bone marrow mesenchymal stem cells expanded in autologous serum or fatal bovine serum. Int J Dev Biol..

[34] Mizuno N, Shiba H, Ozeki Y, Mouri Y, Niitani M, Inui T, Hayashi H, Suzuki K, Tanaka S, Kawaguchi H, Kurihara H (2006). Human autologous serum obtained using a completely closed bag system as a substitute for foetal calf serum in human mesenchymal stem cell cultures. Cell Biol Int..

[35] Stute N, Holtz K, Bubenheim M, Lange C, Blake F, Zander AR (2004). Autologous serum for isolation and expansion of human mesenchymal stemcells for clinical use. Exp Hematol..

[36] Shahdadfar A, Frønsdal K, Haug T, Reinholt FP, Brinchmann JE (2005). In vitro expansion of human mesenchymal stem cells: choice of serum is a determinant of cell proliferation, differentiation, gene expression, and transcriptome stability. Stem Cells..

[37] Tateishi K, Ando W, Higuchi C, Hart DA, Hashimoto J, Nakata K, Yoshikawa H, Nakamura N (2008). Comparison of human serum with fetal bovine serum for expansion and differentiation of human synovial MSC: potential feasibility for clinical applications. Cell Transplant..

[38] Kuznetsov SA, Mankani MH, Robey PG (2000). Effect of serum on human bone marrow stromal cells: ex vivo expansion and in vivo bone formation. Transplantation..

[39] Poloni A, Maurizi G, Rosini V, Mondini E, Mancini S, Discepoli G, Biasio S, Battaglini G, Felicetti S, Berardinelli E, Serrani F, Leoni P (2009). Selection of CD271+ cells and human AB serum allows a large expansion of mesenchymal stromal cells from human bone marrow. Cytotherapy..

[40] Le Blanc K, Samuelsson H, Lönnies L, Sundin M, Ringdén O (2007). Generation of immunosuppressive mesenchymal stem cells in allogeneic human serum. Transplantation..

[41] Kocaoemer A, Kern S, Kluter H, Bieback K (2007). Human AB serum and thrombin-activated platelet-rich plasma are suitable alternatives to fetal calf serum for the expansion of mesenchymal stem cells from adipose tissue. Stem Cells..

[42] Dominici M, Le Blanc K, Mueller I, Slaper-Cortenbach I, Marini F, Krause D, Deans R, Keating A, Prockop DJ, Horwitz E (2006). Minimal criteria for defining multipotent mesenchymal stromal cells. The International Society for Cellular Therapy position statement. Cytotherapy..

[43] Zeddou M, Relic B, Malaise MG (2014). Umbilical cord fibroblasts: Could they be considered as mesenchymal stem cells?. World J Stem Cells..

[44] Nagamura-Inoue T, He H (2014). Umbilical cord-derived mesenchymal stem cells: Their advantages and potential clinical utility. World J Stem Cells..

[45] Lohmann M, Walenda G, Hemeda H, Joussen S, Drescher W, Jockenhoevel S, Hutschenreuter G, Zenke M, Wagner W (2012). Donor age of human platelet lysate affects proliferation and differentiation of mesenchymal stem cells. Plos One..

[46] Lange C, Brunswig-Spickenheier B, Eissing L, Scheja L (2012). Platelet lysate suppresses the expression of lipocalin-type prostaglandin D2 synthase that positively controls adipogenic differentiation of human mesenchymal stromal cells. Exp Cell Res..

[47] Gruber R, Karreth F, Kandler B, Fuerst G, Rot A, Fischer MB, Watzek G (2004). Platelet-released supernatants increase migration and proliferation, and decrease osteogenic differentiation of bone marrow-derived mesenchymal progenitor cells under in vitro conditions. Platelets..

[48] Abdelrazik H, Spaggiari GM, Chiossone L, Moretta L (2011). Mesenchymal stem cells expanded in human platelet lysate display a decreased inhibitory capacity on T- and NK-cell proliferation and function. Eur J Immunol..

[49] Copland IB, Garcia MA, Waller EK, Roback JD, Galipeau J (2013). The effect of platelet lysate fibrinogen on the functionality of MSCs in immunotherapy. Biomaterials..

